# Sensorimotor functioning changes in response to global exercise versus handwriting upper limb exercise training in Parkinson’s disease, results from a phase II randomised controlled trial

**DOI:** 10.1371/journal.pone.0309217

**Published:** 2024-08-29

**Authors:** Íbis Ariana Peña de Moraes, Johnny Collett, Talita Dias da Silva, Marloes Franssen, Surabhi Mitta, Paweł Zalewski, Andy Meaney, Derick Wade, Hooshang Izadi, Charlotte Winward, Carlos Bandeira de Mello Monteiro, Helen Dawes

**Affiliations:** 1 National Institute for Health and Care Research, Exeter Biomedical Research Centre, College of Medicine and Health, University of Exeter, Exeter, United Kingdom; 2 Postgraduate Program in Rehabilitation Sciences, Faculty of Medicine, University of São Paulo, São Paulo, São Paulo, Brazil; 3 College of Medicine, University City of São Paulo, São Paulo, São Paulo, Brazil; 4 Movement Science Group, Oxford Brookes University, Oxford, United Kingdom; 5 Postgraduate Program in Medicine - Cardiology at Escola Paulista de Medicina, Federal University of São Paulo, São Paulo, São Paulo, Brazil; 6 Nuffield Department of Orthopaedics, Rheumatology and Musculoskeletal sciences, University of Oxford, Oxford, United Kingdom; 7 Department of Psychology, University of Buckingham, Buckingham, United Kingdom; 8 Department of Exercise Physiology and Functional Anatomy, Ludwik Rydygier Collegium Medicum in Bydgoszcz Nicolaus Copernicus University in Torun, Bydgoszcz, Poland; 9 Department of Experimental and Clinical Physiology, Laboratory of Centre for Preclinical Research, Warsaw Medical University, Warsaw, Poland; 10 Oxford Centre for Enablement, Oxford University Hospitals, Oxford, United Kingdom; 11 Department of Mechanical Engineering and Mathematical Sciences, Oxford Brookes University, Oxford, United Kingdom; 12 Oxford Allied Health Professions Research Unit, John Radcliffe Hospital, Oxford, United Kingdom; 13 Postgraduate Program in Physical Activity Sciences, School of Arts, Science and Humanities of University of São Paulo, São Paulo, São Paulo, Brazil; 14 Department of Clinical Neurology, University of Oxford, Oxford, United Kingdom; First Affiliated Hospital of Dalian Medical University, CHINA

## Abstract

**Introduction:**

People with Parkinson’s disease (PwPD) present motor alterations which can impact daily life tasks that require speed and/or accuracy of movement.

**Objective:**

A sub analysis of NCT01439022, aiming to estimate the extent to which two different exercise training protocols (global and handwriting upper limb exercise training) impact reaction time, travel speed, and accuracy in PwPD.

**Methods:**

Seventy PwPD, right-side dominant were randomised 1:1 into two six-month training protocol groups; 35 PwPD performed global exercise training and 35 performed specific training (handwriting upper limb exercise movements). Assessments of speed-accuracy and trade-off were carried out at baseline, after 3 and 6 months of training, and at a 12-month follow-up. The current study used data from a previous publication of a randomised controlled trial that included a 6-month self-managed community exercise programme for PwPD. For the present study we included only the participants who completed the Fitts’ task during the baseline assessment.

**Results:**

In the upper limb assessments, no main effects were found for the number of touches, but the exercise group showed a marginal increase over time on the left side. Error averages on the left side decreased significantly for the exercise group from baseline to 6 and 12 months. The exercise group also presented a lower Error CoV and the Reaction Time CoV increased on the right side. Significant findings for Fitts r on the left side indicated lower values for the exercise group, with improvements continuing at 12 months.

**Conclusion:**

We report the potential of global exercise interventions to facilitate improvements in reaction time and travel speed, as well as other motor control metrics, with lasting effects at 12 months, particularly on the non-dominant side.

## Introduction

Parkinson’s disease (PD) is a progressive neurodegenerative disorder defined by the loss of dopaminergic neurons in the substantia nigra and the development of Lewy bodies in the surviving neurons [1]. People with Parkinson’s disease (PwPD) present cardinal features of bradykinesia, rigidity, tremor, and postural instability, coupled with gradual symptom progression [1, 2], and alterations in qualitative aspects of information processing [3]. Thus, PwPD present difficulties resolving conflicts during action selection and, in particular, in suppressing conflicting responses using strategies that prioritise speed or performance accuracy [4].

The influence of responses triggered by irrelevant information on goal-directed behaviour is largely determined by the need to respond quickly or accurately to a stimulus. Emphasising speed results in quicker responses but raises the probability of errors, whereas emphasising accuracy reduces errors but slows down responses [5]. This trade-off between movement speed and accuracy is formalised in Fitts’ law, which states that movement time relates linearly to the index of difficulty (ID), quantifying task difficulty in aiming tasks [6]. According to Sakurada et al. [7], Fitts’ law is a well formulated law of human movement, which describes the time required to move a pointer as quickly and accurately as possible between two targets as a function of the width of the targets and the distance between them. Moreover, another difficulty of PwPD that can influence speed and accuracy is the bilateral action tremor, which includes both postural and kinetic tremors of the hands. Action tremors most commonly involve the upper extremities in a symmetric manner [8].

PwPD often struggle to maintain rhythmic movements, such as finger tapping, and exhibit deficits in timing, specifically in the regulation of force and time parameters, rather than only in force production, leading to lack of accuracy and speed [6]. As the disease progresses, difficulties in performing specific activities become more pronounced, with writing difficulties being a common early complaint in PwPD [9]. Writing is a complex functional activity that combines both automated and controlled processes. Micrographia, a clinical sign defined by impaired fine motor skills, manifests primarily as a reduction in writing amplitude, either progressive or stable. This is often one of the earliest indicators of Parkinson’s disease, affecting approximately 75% of PwPD [10–12]. Although writing problems seem to respond well to dopaminergic medication, improvements resulting from medication are mainly found in travel speed and, often to a lesser extent, in writing size [13]. In addition to dopaminergic medication, rehabilitation has been found to improve motor function in PwPD, offering significant benefits in reducing the severity of motor signs and enhancing quality of life [14, 15].

Considering PwPD rehabilitation, some interventions prioritise global training, such as aerobic exercise, aquatic therapy, dance, strength/resistance, balance training, and endurance training [14, 16, 17], while others focus on upper limb specific training, such as hand exercises and writing activities [17, 18]. In previous studies we found improvement in motor symptoms in favour of the exercise group [19] and improvements in writing amplitude in favour of the handwriting group [10]. However, there is still a gap in the knowledge: “What is the influence of global and specific movements in the improvement in accuracy and speed?”. The answer to this question would help clinicians to understand how to design better clinical treatments, in order to improve the accuracy and speed of PwPD, and thus improve their functional movements.

To answer this question, we conducted a secondary analysis of the trial data (NCT01439022), comparing upper limb specific handwriting training and global exercise training (different exercises). Both groups were evaluated by a computer task that uses the upper limbs, through analysis of the reaction, movement time, and errors, together with the relationship between speed and accuracy based on Fitts’ law. The goal of our study was to verify which training protocol (global or specific movement) influences speed and accuracy in PwPD. We hypothesised that PwPD would show improvements in speed and accuracy after both training protocols during the assessments and follow-up, however, given that the assessment consisted of an upper limb task, we expected the handwriting-specific exercise group to demonstrate superior performance compared to the global exercise group.

## Methods

### Design

**Fig 1:**
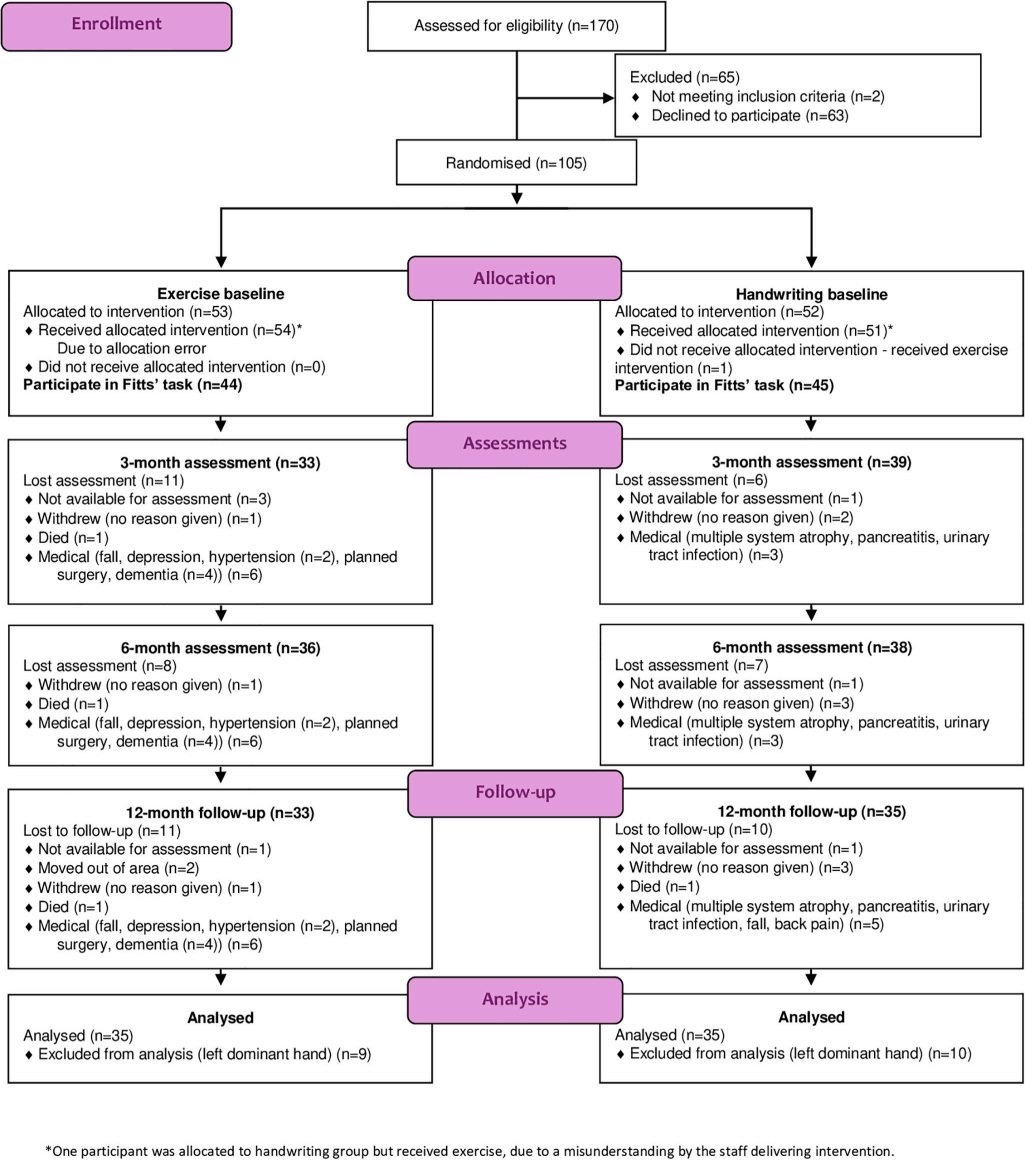
CONSORT flow diagram.

This report includes measures of the speed-accuracy trade-off obtained from a two-arm parallel single-blind phase II randomised controlled trial of exercise (RCT). The primary study results can be found in Collett et al. [19, 20] and Mavrommati et al. [21]. The trial received ethical approval (National Research Ethics Service (NRES): 11/SC/0267) and was registered with ClinicalTrials.Gov (NCT01439022). Full reporting of the trail is according to Consolidated Standards of Reporting Trials (CONSORT) guidelines [22, 23], which are presented in the Supporting Information (S1 Checklist and S1 File). The majority of the methodology used in this secondary analysis was the same as for the RCT (Fig 1). Written informed consent was obtained from all subjects involved in the study and the collections were performed after the research participant signed the Informed Consent Form. Data were collected from December 05, 2011 to August 30, 2013.

Participants were recruited from general practitioner (GP) practices, neurology clinics, and local Parkinson’s UK meetings in the Thames Valley, UK. They were randomly assigned (1:1) to either an exercise group or a handwriting training group using computer-generated randomisation programme, carried out by an independent researcher who was not involved in the recruitment or assessment of participants. The group allocation was concealed from the assessor until the end of the study.

Inclusion criteria were: diagnosis of idiopathic PD, being able to walk ≥100 m [24], and participating in the Fitts’ task during the baseline assessment. Exclusion criteria were: history of an additional prior neurological condition, cardiovascular or orthopaedic problems that limited exercise practice, and being left-hand dominant, so as to avoid the influence of central structures (cerebral laterality) in the motor task.

### Exercise training group

The exercise group sessions took place at community leisure facilities, with monthly visits from a qualified clinical exercise specialist. The exercise program, accessible through a booklet (available upon request via email to jcollett@brookes.ac.uk), consisted of 30 minutes of aerobic training (targeting 55–85% of age-predicted maximum heart rate (220 minus age)) followed by 30 minutes of resistance training. The program spanned 24 weeks, with a total of 48 sessions, customised and performed based on specific protocols. Exercise intensity and progression were monitored during the monthly support sessions. During the initial session, the exercise professional or physiotherapist calibrated the exercise intensity to ensure participants achieved the target heart rate range during the aerobic training. Participants were then instructed on how to adjust speed or resistance in order to maintain this intensity in subsequent sessions. The initial resistance level was selected to enable participants to complete ten repetitions [19, 20].

### Handwriting training group

The handwriting sessions were conducted at participants’ residences, with monthly support visits from the same staff who facilitated the training sessions. The program, provided via handwriting workbooks (available upon request through jcollett@brookes.ac.uk), started with warm-up hand movements, followed by various writing exercises using the dominant hand, it did not involve any movement or exercises for the non-dominant hand. These movements included manipulating putty, attaching pegs to a jar, putting sticks into and removing them from a jar, and catching a ball, each repeated between 3 and 10 times per session. There was no formal customization or progression, as all handwriting group participants followed identical workbooks. However, participants were able to gauge their performance using the pangram "The quick brown fox jumps over the lazy dog," which was practised in every session. Feedback was offered by the clinical professional during the monthly support meetings [19, 20].

### Assessments

Demographic data were collected during the initial assessment. All outcome measures were evaluated at baseline (entry), and after 3 months (midpoint of the intervention), 6 months (end of the intervention), and 12 months (follow-up). The evaluations were consistently performed by the same evaluator, who remained blinded to the participants’ assigned interventions throughout the study. The medication taken by participants was recorded and continued as normal. The duration that a patient remains in the ON or OFF state can vary significantly depending on several factors, including medication dosage, disease progression, and individual response to medication [25]. Therefore, participants adhered to their usual Parkinson’s medication regimen, with assessments conducted during the ON state for those experiencing ON and OFF periods, without control over the state in which the training occurred. The trial incorporated a comprehensive battery of measures to explore the potential impacts of the interventions [19, 20]. Here, we specifically report on the assessment of the speed-accuracy trade-off during an upper limb task.

For sample characterization, we evaluated: (1) Motor performance: 2 minute walk test [26]; Unified Parkinson’s disease rating scale MDS-UPDRS (III) [27]; Timed up and go test [26]; (2) Fitness: Maximal oxygen consumption (VO2max L/min) [28], using a stepwise incremental exercise test [10]; Leg power using a ‘power metre’; Grip strength using a hand-held dynamometer [29]; Health and well-being: Health-related quality of life was measured using the Euro-QOL (EQ5D-5L) [30]; and Short Form Health Survey (SF-36) [31]; Fatigue was self-reported using the Fatigue Severity Scale (FSS) [32]; Body mass index (BMI); and Physical Activity Scale for the Elderly (PASE) [33].

A total of 170 individuals, contacted through one or more of the cited institutions, expressed interest in the study. Of these, 107 were assessed for eligibility, with only 2 not meeting the criteria, resulting in 105 randomised participants. Of these, 89 participated in the Fitts’ task. One participant, who was supposed to receive the handwriting intervention, mistakenly received the exercise intervention instead. This participant was included in the analysis as part of the exercise group. The allocation error occurred due to a misunderstanding by the staff delivering the intervention and was discovered after the intervention had been completed.

The participant flow is detailed in Fig 1. Two individuals were excluded after randomisation, one from each group, as they no longer met the eligibility criteria following a revised or additional diagnosis (Lewy body dementia, multiple system atrophy). Most medical-related exclusions occurred at the 3-month assessment, leading to participant dropout. Among those who dropped out, eight experienced serious adverse events, all of which were deemed unrelated to either intervention. In the exercise group, six participants reported adverse events: two had falls resulting in hospitalisation, one died, and three had planned surgeries. In the handwriting group, two participants experienced adverse events: one had acute pancreatitis, and the other had a urinary tract infection.

In the exercise group, all discontinuations occurred within the first three months. In the handwriting group, three participants discontinued the intervention for medical reasons at the 3-month assessment. Additionally, two serious adverse events were recorded that did not result in discontinuation: a fall and a death, both of which occurred during the follow-up period after the intervention had ended. There were no intervention-related adverse events in the handwriting group.

### Fitts’ task

**Fig 2:**
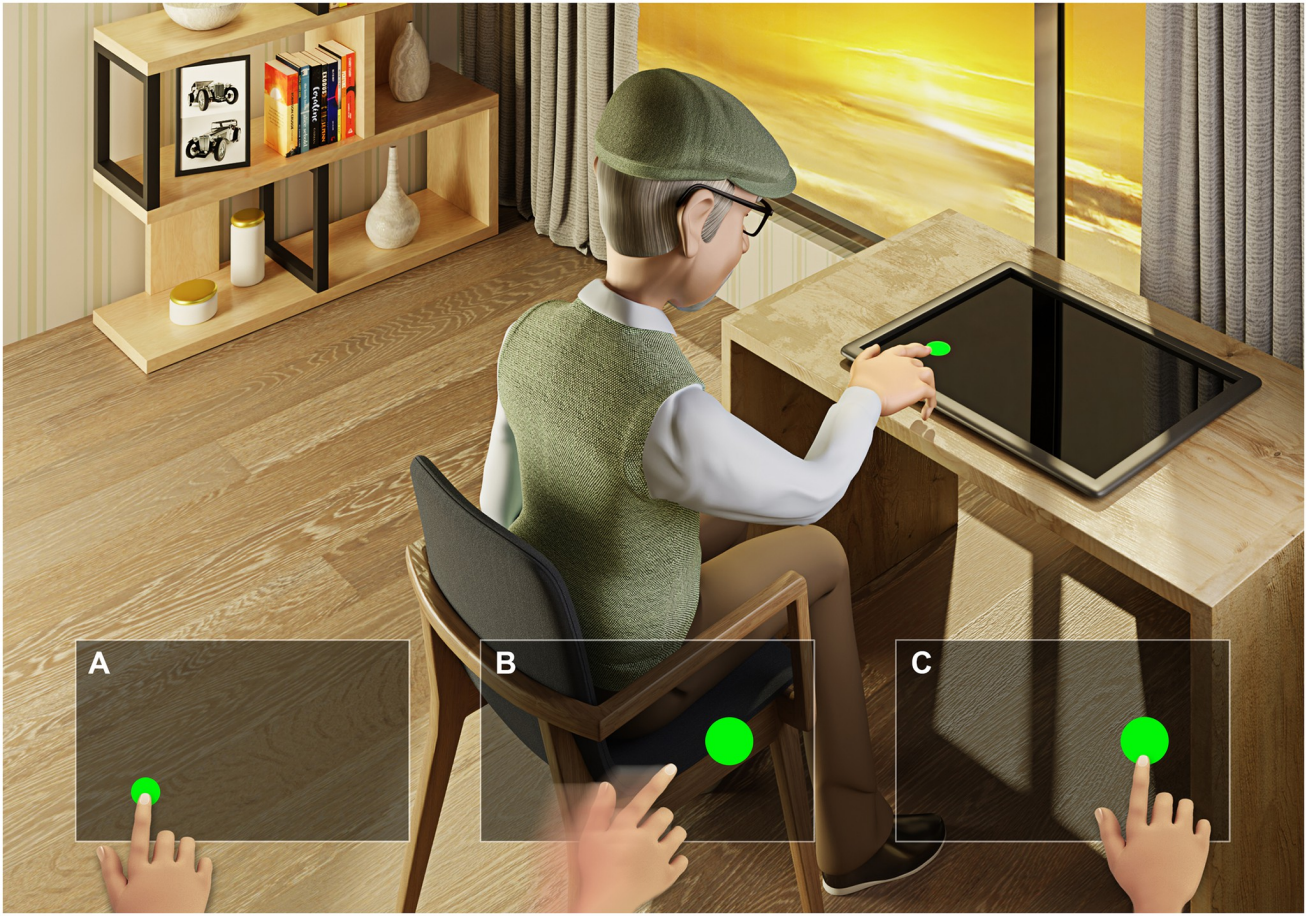
Representative design of the Fitts’ task using the touchscreen interface. A- example of the first trial with a small target; B- moving to a bigger target; C- touching in the second trial with a bigger target.

The task was delivered, and the measurements derived using a bespoke program on a touch screen personal computer. The task involved the participant placing their index finger on a solid circle displayed on the screen, after which a new circle (target) appeared, and participants were required to move their finger to touch the next target. A trial included 24 targets and the time between targets, subsequent target position, and size of the target were random (3 potential target sizes 15, 20, 25mm). In total, 3 trials were performed for each hand after the participant had completed a practice trial (Fig 2).

In Fitts’ law, considered as the speed-accuracy trade-off, the relationship between speed and accuracy can be described by a mathematical equation, such that there is a log-linear relationship between movement time and task difficulty, with more time required to reach targets of smaller sizes due to the increase in accuracy requirements [34]. The result is formulated as follows: MT = a + b × ID; where MT is the average movement time to reach a target, defined as a linear function of the index of difficulty (ID) of the movement, and ID is a function of the distance to be covered, commonly referred to as distance between targets (D), and width (W) of the target to be approached. The ID is expressed as follows: ID = log2(2D/W): the higher the difficulty the slower the movement [7, 34, 35]. Measures obtained were: **(1)** Touches: number of touches made by the participants. **(2)** Error (distance between centre of target and where finger touched, in x and y coordinates). Distance (linear distance between previous and new target), **(3)** Reaction time (time taken to remove finger from previous target after new target appears), **(4)** Travel speed (distance / travel time), in which: travel time is the time taken between removing finger from previous target and touching new target; and Index of difficulty (Shannon–Log (distance / target size +1.2): curve intercept (b0), **(5)** slope (b1), and **(6)** variation (r).

### Data analysis

While the original study aimed to evaluate the feasibility of the exercise program, the present study was a phase II trial, which was not designed to determine efficacy. The sample size was based on the estimated effect on 2-minute walk distance and did not consider effects on handwriting outcomes. The Index of difficulty (x) was plotted against travel time (y) to determine the Fitts’ law relationship. Data were then averaged over all 3 trials and the coefficient of variation was calculated (CoV = mean / standard deviation). For the current analysis, a protocol approach was used, whereby to be included individuals were required to have completed the Fitts’ motor task in the baseline assessment and to be right-hand dominant. Descriptive statistics were calculated for demographic characteristics and compliance data. The independent samples t-test or χ^2^ test was used to assess differences between group means and frequencies at baseline using the Statistical Product and Service Solutions (SPSS; IBM, Chicago, IL), version 28.0. For outcome data the linear mixed models (LMM) procedure of SAS V.9.4 was used to determine the mean changes in curve intercept (b0), slope (b1), and variation (r), as response variables, according to two intervention regimes (exercise and handwriting) and four repeated measures (assessments: baseline, 3-month, 6-month, and 12-month follow-up). In addition, we ran the RM-MANOVA with factors 2 Groups (between—Handwriting and Exercise) by 4 Assessments (within—baseline, 3 months, 6 months, and 12 months) using SPSS, in order to show a head-to-head comparison, the effect sizes, F-value, degrees of freedom, and variance are included as supporting information (S2 File). Values of p<0.05 were considered significant. All data from this research are publicly available [36].

## Results

Recruitment, randomisation, and participant flow are available in the study of Collett et al. [19, 20], that randomised 105 participants. Fitts’ law data were assessed in 89 of these participants (Exercise group n = 44, Handwriting group n = 45). We chose to keep only the right-hand dominant participants, totalling 70 participants, 35 in each group. Table 1 presents the demographic data. No statistical differences were found between the groups, demonstrating sample homogeneity.

**Table 1:** Demographic data.

	Exercise	Handwriting	p-value
**Demographics**	n = 35	n = 35	
Age (years)	65.5±7.6	67.2±6.9	0.789
Sex (M:F)	20:15	16:19	0.339
Time since diagnosis	4.8±3.7	5.5±4.6	0.105
**Motor symptoms**			
Two-minute walk test (m)	148.2±19.1	135.1±22.9	0.322
UPDRS part III	15.9±10.5	18.7±10.8	0.933
TUG (s)	9.28±1.8	10.3±2.3	0.252
**Fitness**			
VO2 (L/min)	22.2±7.2	18.9±5.9	0.139
Leg power (W)	144.9±60.8	123.6±55.6	0.487
Grip strength (W)	31.5±9.3	29.3±9.8	0.509
**Health and well-being**			
EQ5D-5L	73.4±15.5	71.7±21.0	0.220
SF36	70.9±17.4	68.6±18.9	0.607
FSS	3.5±1.5	3.8±1.6	0.887
BMI	27.1±4.8	27.1±4.4	0.326
PASE	65.4±35.9	68.6±35.4	0.772

Mean±SD; F: Female; M: Male; UPDRS III: Movement Disorder Society Unified Parkinson’s Disease Rating Scale part III; TUG: Timed up and go test; VO2, oxygen consumption; EQ5D-5L: index score of the Euro-QOL EQ5D-5L; SF-36: Short Form (36 item) Health Survey, physical and mental subscores; FSS: Fatigue Severity Scale; BMI: Body Mass Index (weight (kg)/(height (m)2); PASE: Physical Activity Scale for the Elderly.

### Outcomes

**Fig 3:**
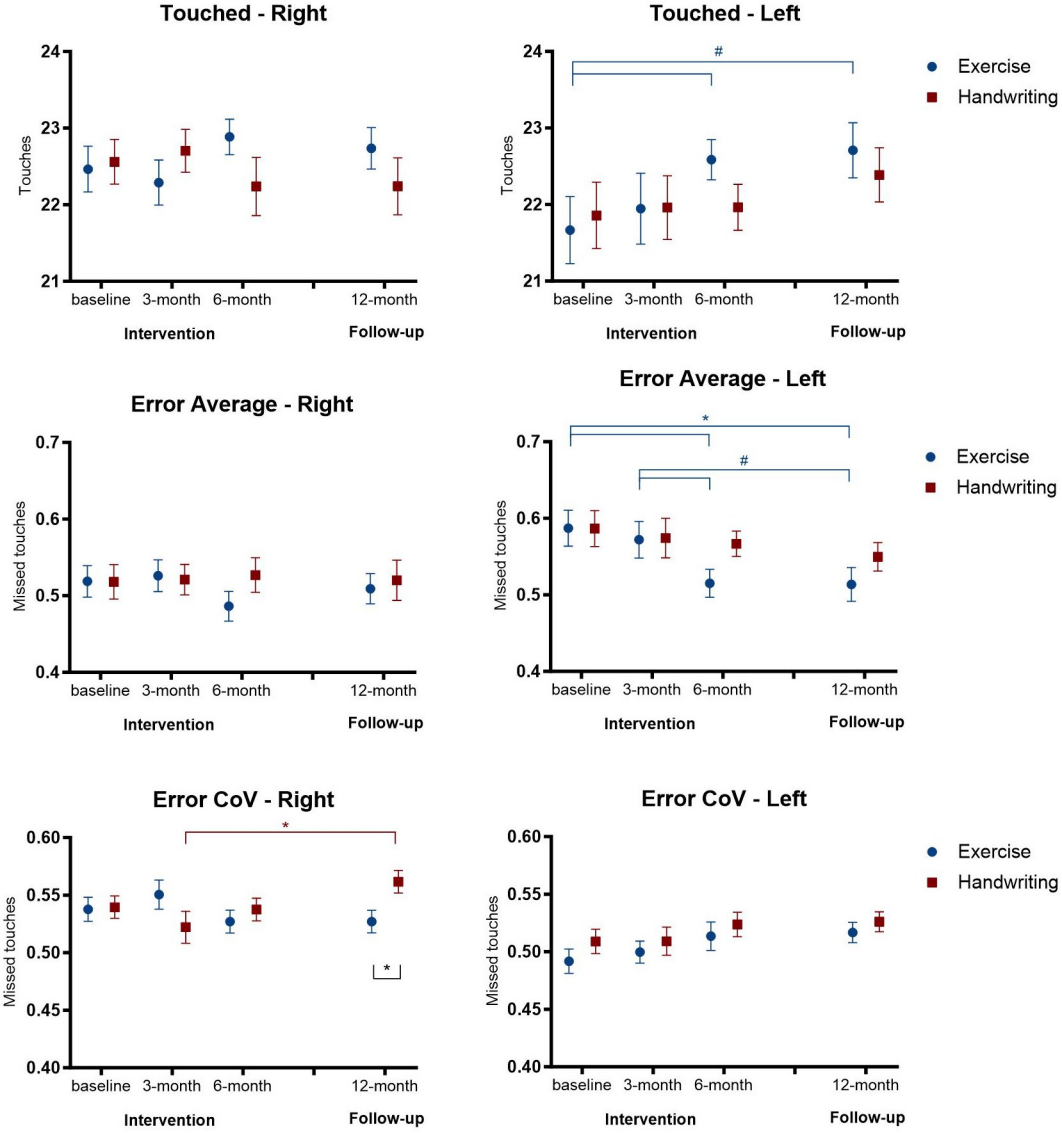
Representation of mean and standard error of touches, error average, and error CoV in the four assessments in the exercise and handwriting groups. * p < 0.05 # p > 0.05 < 0.08.

No main effects were found for the Touches variable; however, post hoc comparisons showed a marginal difference for the exercise group on the left side, from the baseline assessment to the 6-month assessment (p = 0.074), and to the 12-month follow-up (p = 0.055). This result indicates a trend of an increasing number of touches in the final intervention and follow-up assessments (Table 2 and Fig 3).

**Table 2:** Representation of means and standard deviations (SD) of all variables in the study.

		Baseline		3-month assessment		6-month assessment		12-month follow-up	
		Exercise	Handwriting	Exercise	Handwriting	Exercise	Handwriting	Exercise	Handwriting
Variable		Mean±SD	Mean±SD	Mean±SD	Mean±SD	Mean±SD	Mean±SD	Mean±SD	Mean±SD
**Touches**	Right	22±1.8	23±1.6	22±1.3	22±1.4	22±1.4	22±1.3	22±1.6	22±1.8
**Error Ave**		0.51±0.13	0.51±0.12	0.51±0.10	0.51±0.09	0.49±0.10	0.51±0.08	0.51±0.11	0.50±0.12
**Error CoV**		0.55±0.06	0.54±0.05	0.53±0.06	0.52±0.07	0.53±0.04	0.54±0.05	0.54±0.05	0.56±0.04
**RT Ave**		373±807	366±585	375±590	367±576	365±511	365±528	361±608	362±421
**RT CoV**		0.19±0.09	0.17±0.07	0.22±0.10	0.18±0.08	0.21±0.11	0.17±0.06	0.19±0.09	0.18±0.06
**Travel speed Ave**		0.04±0.01	0.04±0.01	0.03±0.01	0.04±0.01	0.03±0.01	0.04±0.01	0.03±0.01	0.03±0.01
**Travel speed CoV**		0.3±0.1	0.29±0.05	0.31±0.07	0.28±0.05	0.29±0.04	0.27±0.04	0.31±0.08	0.31±0.04
**Fitts r**		0.6±0.02	0.59±0.17	0.59±0.18	0.63±0.16	0.62±0.16	0.66±0.14	0.59±0.17	0.60±0.16
**Fitts intercept**		229±1091	214±2.149	283±1937	246±913	280±2314	228±982	299±3089	235±1500
**Fitts slope**		283±1168	354±3.252	270±1639	262±1309	269±2134	275±647	294±1993	353±2482
**Touched**	Left	22±24	22±16	22±23	22±20	22±16	22±12	22±17	22±19
**Error Ave**		0.58±0.14	0.56±0.09	0.56±0.12	0.56±0.11	0.54±0.11	0.55±0.07	0.52±0.10	0.54±0.10
**Error CoV**		0.50±0.06	0.51±0.05	0.51±0.05	0.51±0.06	0.50±0.06	0.52±0.05	0.51±0.04	0.52±0.04
**RT Ave**		383±706	380±605	377±611	368±641	373±539	375±494	361±639	369±416
**RT CoV**		0.22±0.10	0.20±0.10	0.19±0.08	0.19±0.08	0.21±0.08	0.21±0.11	0.20±0.10	0.21±0.11
**Travel speed AVE**		0.03±0.01	0.03±0.01	0.03±0.01	0.03±0.01	0.03±0.01	0.03±0.01	0.03±0.01	0.03±0.01
**Travel speed CoV**		0.33±0.10	0.31±0.08	0.29±0.05	0.28±0.03	0.31±0.05	0.27±0.03	0.33±0.08	0.29±0.08
**Fitts r**		0.62±0.15	0.63±0.12	0.60±0.14	0.69±0.10	0.62±0.14	0.68±0.13	0.54±0.19	0.63±0.22
**Fitts intercept**		241±1044	239±1088	267±1182	246±1081	280±1492	226±1139	377±2788	278±1944
**Fitts slope**		292±853	329±1872	290±1067	303±824	313±1141	309±718	284±1723	298±1331

Considering the Error average, a main effect was found for the left side for Assessments (p = 0.037). Post-hoc comparisons show a decrease in the error average among assessments only in the left side exercise group, from the baseline assessment to the 6-month assessment (p = 0.013) and to the 12-month follow-up (p = 0.017), and a marginal difference from the 3-month assessment to the 6-month assessment (p = 0.070), and to the 12-month follow-up (p = 0.075).

An interaction was found between Groups and Assessment (p = 0.050) with Error CoV. The post hoc comparisons identified that the Error CoV right side was lower in the exercise group in the 12-month follow-up (p = 0.034), and in the handwriting group there was an increase from the 3-month assessment to the 12-month follow-up (p = 0.013).

No effect was found for the variables Reaction Time Average and Travel Speed CoV. Considering Reaction Time CoV, a main effect was found for Groups (p = 0.028) on the right side, indicating that the exercise group had a higher Reaction time CoV than the handwriting group. Furthermore, the exercise group showed a significant increase from the baseline assessment to the 3-month assessment (p = 0.037).

**Fig 4:**
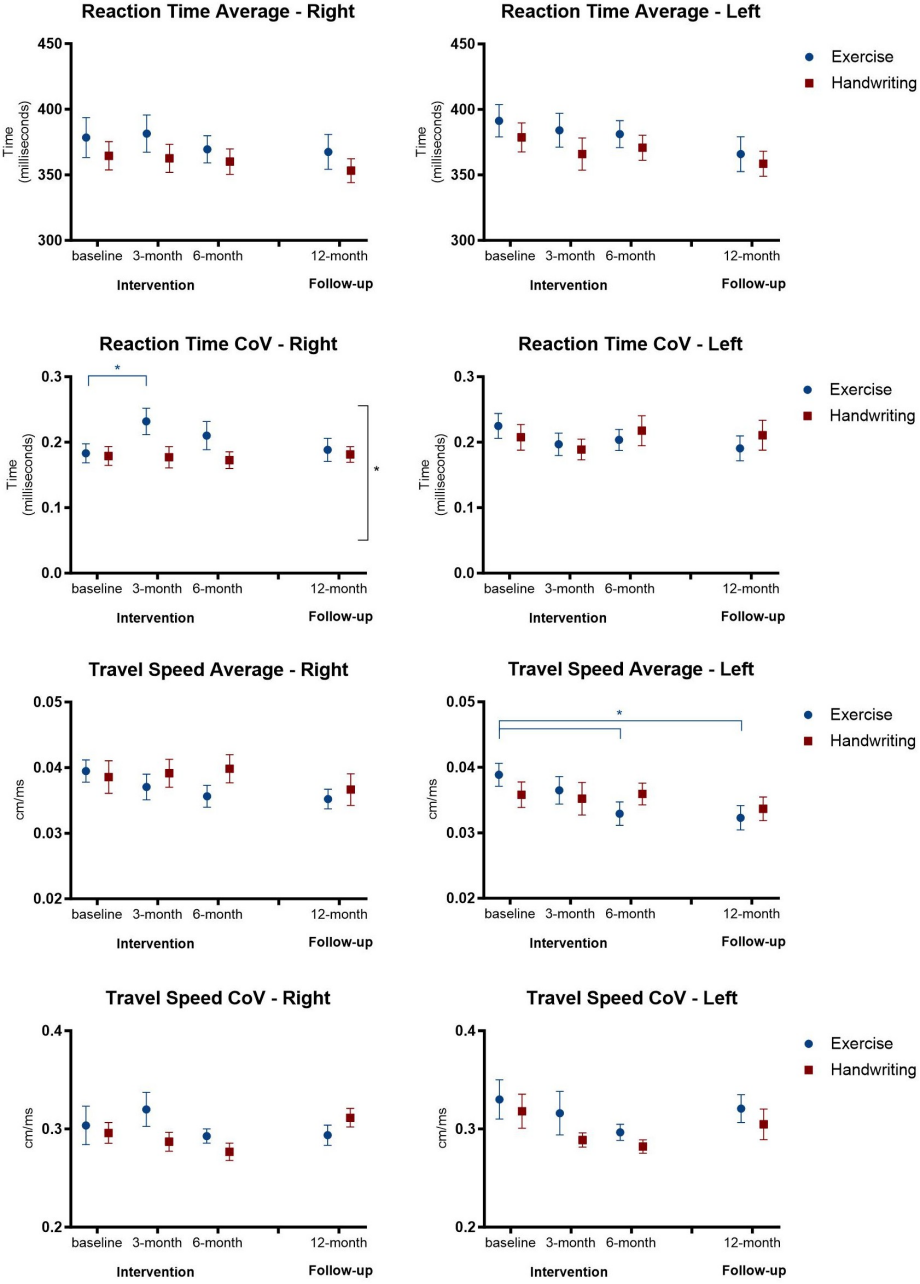
Representation of mean and standard error of reaction time average and CoV, and of travel speed average and CoV in the four assessments in the exercise and handwriting groups. * p < 0.05.

No main effects were found for the Travel speed CoV, however, post hoc comparisons showed a significant difference for the exercise group on the left side, from the baseline assessment to the 6-month assessment (p = 0.020), and to the 12-month follow-up (p = 0.015), indicating a decrease in the final assessments (Fig 4).

**Fig 5:**
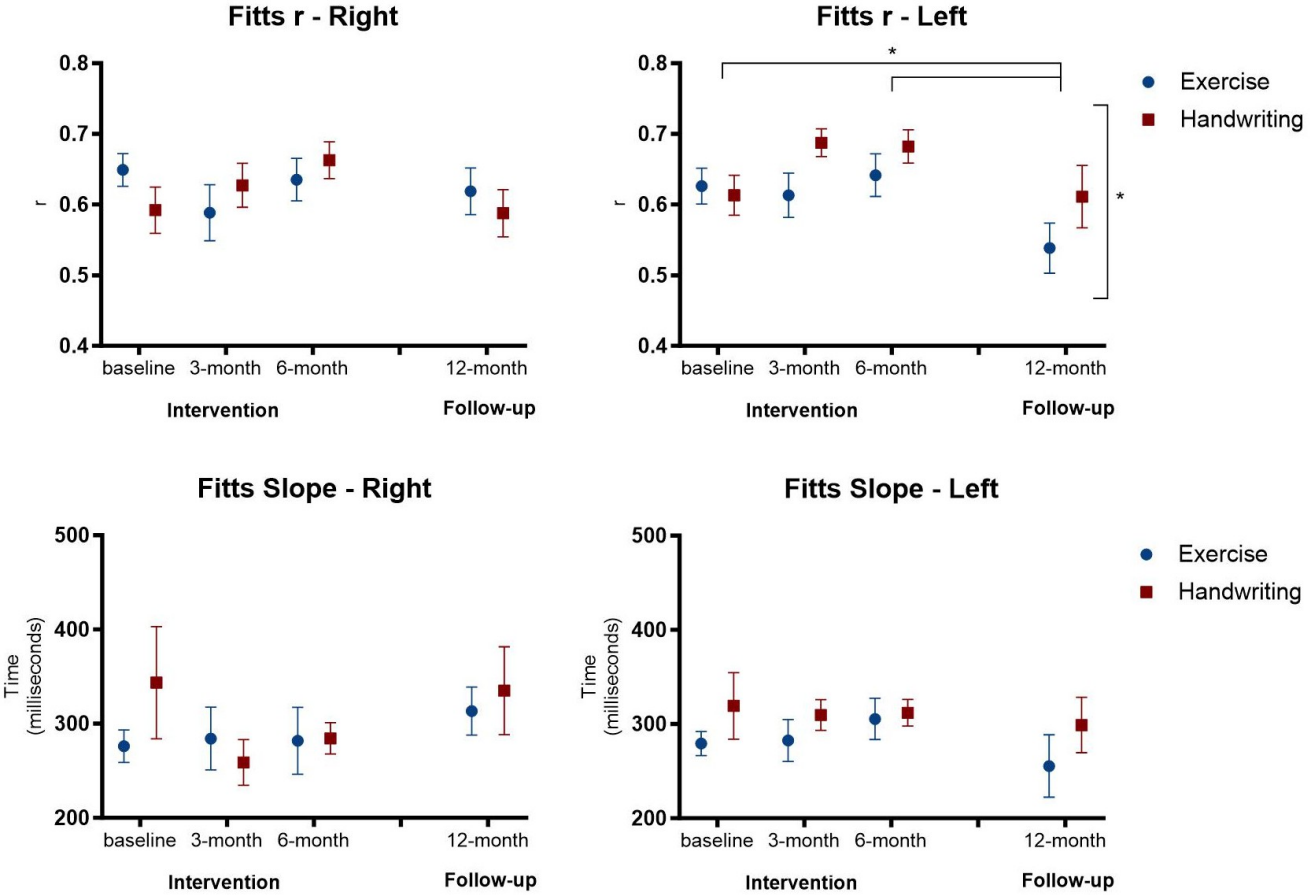
Representation of mean and standard error of Fitts r, slope, and intercept in the four assessments in the exercise and handwriting groups. * p < 0.05.

Although there were no significant main interactions between Groups and Assessments, there were significant findings for the left side of the body regarding Fitts r, for Groups (p = 0.042) and Assessments (p = 0.026), showing a lower Fitts r on the left side for the Exercise group. In addition, this group showed a lower Fitts r in the 12-month follow-up compared to the baseline assessment (p = 0.037) and 6-month assessment (0.018). No effect was found for the Fitts slope (Fig 5).

**Fig 6:**
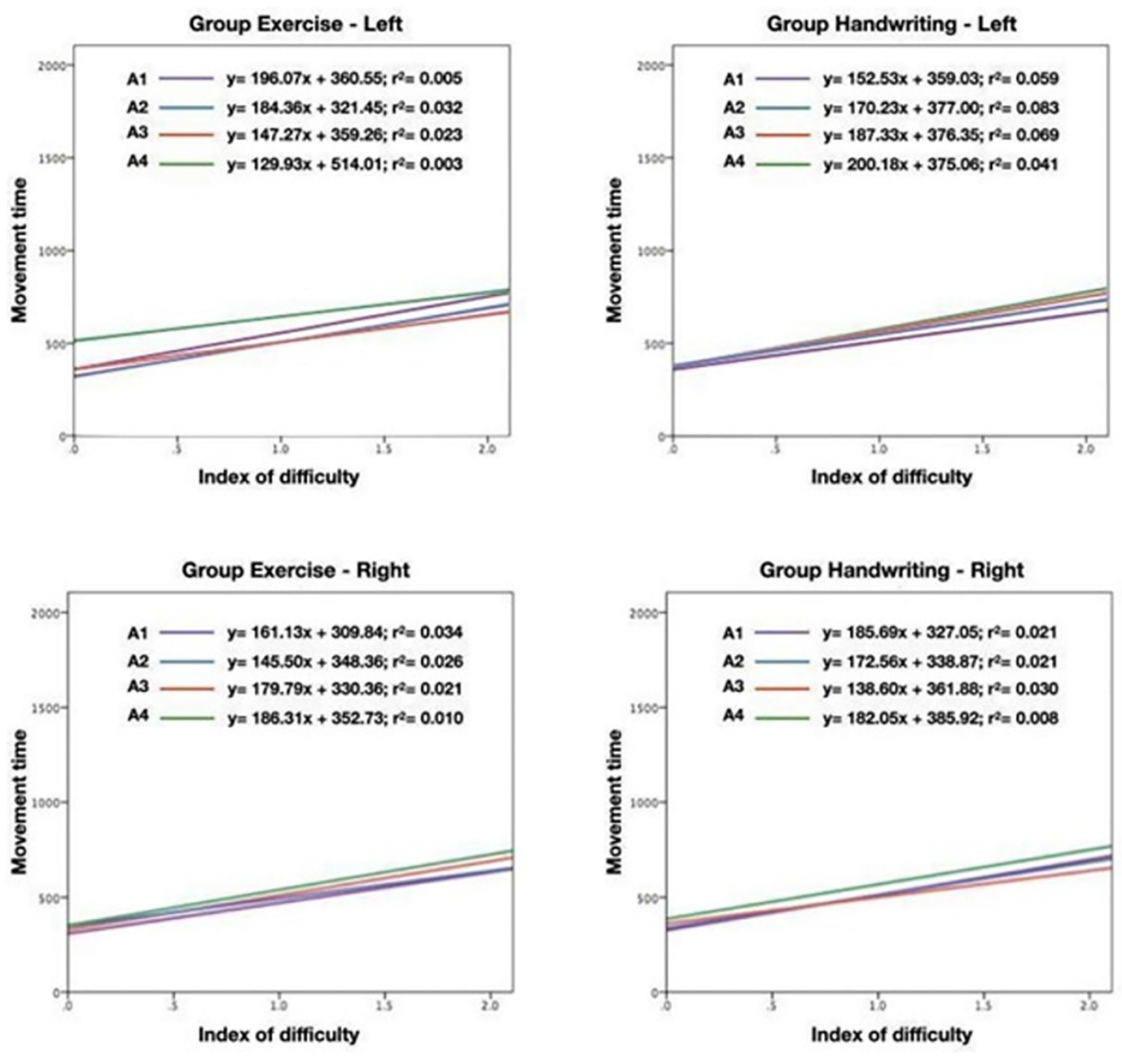
Representation of movement time according to difficulty indices in the four assessments (A1-A4) in the exercise and handwriting groups. A1: Baseline assessment; A2: 3-month assessment; A3: 6-month assessment; A4: 12-month follow-up.

Fig 6 presents the progression of movement time increases in relation to the difficulty indices, showing no differences between groups and assessments.

## Discussion

The global exercise group showed general improvements in the upper limbs in reaction time and travel speed, with improvement in number of touches and accuracy in the non-dominant side (left) over the training period, in the 6-month intervention and follow-up period. Both the handwriting and global exercise groups demonstrated improvements in speed and the ratio (r) of the Fitts curve (Fig 6) after the intervention. Our results demonstrate the potential for improvements in reaction time, travel speed, and other motor control metrics from global exercise interventions, with lasting effects over 12 months particularly on the non-dominant side. Whilst testing was focused on the upper limbs, our findings have implications for furthering understanding of the mechanisms of global exercises that improve the speed-accuracy trade-off, and their role in functional daily tasks which require upper limb accuracy.

### 1- Global and Handwriting exercises

When comparing global (exercise) and specific practice (Handwriting) we observed that handwriting training did not improve any parameters evaluated through Fitts’ Law. We can speculate that the handwriting intervention was not able to enhance skills sufficiently for speed or accuracy improvement. Different studies presented benefits from exercise to upper limb function using task-oriented training [37], virtual reality [38], and goal-oriented interventions [18]. A randomised controlled trial [39] using fine-tuned control of force, finger independence, finger coordination, and motor sequence performance showed that an intensive, task specific, home-based dexterity program significantly improved fine motor skills in PwPD, however, we did not find important improvement in the handwriting training group in our study. Although PwPD moved more slowly than control participants and with less accuracy [6], velocity and acceleration are considered to be the main kinematic features of handwriting as well as of Fitts’ task (i.e., leading us to hypothesise that handwriting training could improve performance in a speed and accuracy task). We can speculate that Fitts’ law is a complex process requiring cognitive, perceptual, and fine motor abilities and that the handwriting training proposed was not sufficient to provide benefits to PwPD. Thomas, Lenka and Kumar [9] stated that considering the function of the basal ganglia in motor learning, it can be postulated that PwPD would have trouble improving fine motor tasks. The study of Mazzoni et al. [40] also supports the idea that PwPD move more slowly than normal individuals, even though their ability to move normally is fully preserved. The higher frequency of slower movements generated by patients, indicates a “choice” (albeit implicit) of slower movements (i.e., PwPD move slowly when the energetic demands of a movement task increase) rather than an inability to execute fast accurate movements. Thus, our results showed no benefit to speed and accuracy from the handwriting intervention, in agreement with statements from other authors [40] that speed selection abnormality was attributable to increased sensitivity to the energetic demands of the movements and not to a change in the speed–accuracy trade-off.

### 2- Body laterality using global movements

Considering the influence of global training (exercise) and specific practice (Handwriting) on improvement in speed and accuracy of movement in PwPD, we found some interesting results for laterality. Persistent asymmetry is one of the main motor symptoms that distinguish PD from other syndromes, since few PwPD show bilateral symmetry [41]. According to Riederer et al. [42], there is increasing evidence that lateralisation is an important symptom in PwPD, due to a complex interplay of hereditary and environmental factors that are reflected in the concept of dominant hemispheres and handedness, and in specific susceptibilities of neuronal subpopulations within the substantia nigra.

Considering laterality, our results showed greater improvement in performance for the global exercise group in travel speed average and error average, and maintenance of performance after 6 months and retention after 12 months (follow-up) only in the non-dominant arm. Exercise possibly influences speed and accuracy of movement due to the improvement in the overall motor condition provided by global exercise, mainly in the arm with more difficulty.

Among the treatments for PwPD, global exercise is currently an area receiving substantial research attention, as investigators seek interventions that may modify the progression of the disease through decreases in bradykinesia [43] and increases in strength [44]. It has been shown that exercise with global movements is a legitimate disease-modifying therapeutic option, that contributes to behavioural recovery and neurochemical sparing in PwPD [45]. According to Oliveira et al. [46], the improvement in bradykinesia and muscle strength with global exercise is an important intervention aim in PwPD. Muscle weakness and slowness of movement are disabling symptoms in PwPD and should be focused on during rehabilitation. Muscle weakness has been related to bradykinesia because they share common pathophysiological mechanisms, and are also responsible for functional performance declines [47]. According to David et al. [43], there was a significant decrease in bradykinesia, with an increase in muscle strength using global exercise with progressive resistance training in PwPD, as well as a reduction in agonist/antagonist co-contraction. Considering our results that observed benefits with global exercise practice only in the non-preferential arm, we can speculate that muscle weakness and slowness of movement, disabling symptoms in PwPD [46], could be more strongly presented in the non-dominant arm and that this arm was beneficially altered with global exercise, which was responsible for the improvement in performance.

Another speculation that could support the positive influence of global exercise in the non-functional arm is the difficulty of the task considering laterality. Difficulty in task practice (i.e., specific practice) depends on the individual’s current experience and when performance meets or exceeds the learner’s expectations, it should result in steady improvements during practice [48]. However, PwPD present differences in bilateral function, with more specific ability in the dominant arm, and this could interfere negatively in accurate tasks in the non-dominant arm. Scharoun et al. [49] aimed to analyse which upper limb is more vulnerable in PwPD. Comparisons indicated that when the non-dominant hand is affected by PD motor symptoms, there is superior performance in the dominant hand in tasks that require precision. The evident improvement found only in the performance of the non-dominant hand with global exercise, may be due to the fact that the dominant side already presented good performance in speed and accuracy at the first moment (first assessment), so no modifications were likely to be observed after training with either global or specific training. Thus, practice with global or specific training did not influence the dominant speed and accuracy ability. However, this improvement was observed in the non-dominant arm in the global exercise group, which can be justified considering that global exercise was able to improve global abilities that provide stability for the task proposed, such as greater motor skills with more muscular recruitment and intersegmental torques, resulting in better muscle coordination [50–52].

### Study limitations and future studies

The current study should be viewed within the limitation that it was not designed to evaluate the handwriting intervention, and thus the sample size, eligibility criteria, intervention, and outcome measures were primarily chosen for the aims of the main study [19]. The current study used data from a prior publication with a randomised controlled trial of a 6-month self-managed community exercise programme for PwPD. For the present study we included only participants who completed the Fitts’ task during the baseline assessment. Although we found some benefits in the global exercise group it should be recognised that the study was not designed to determine efficacy in speed and accuracy and the number of patients was not large, reducing the precision of estimating the size of any benefit. Considering the importance of speed and accuracy in global and specific training we encourage further studies with a protocol using tasks based on Fitts’ law. We can point out some additional limitations: 1) considering that the handwriting sessions took place in the participant’s home and the exercise group sessions at community leisure facilities, we did not assess potential differences in environmental factors, such as participant motivation, task engagement, and reinforcement feedback [53], which could have influenced performance and should be evaluated in future studies. 2) The data were collected 10 years ago, which may limit the relevance of the findings to current clinical practices and advancements in the treatment of Parkinson’s disease. 3) The global exercise group performed different types of training, using both hands, whereas in the handwriting group, only the dominant hand was used. This factor could have influenced the results, as we found better results in the non-dominant hand only for the exercise group. We suggest that future studies should use specific training for both upper limbs.

### Clinical relevance

The global exercise group presented general improvement in reaction time and travel speed, with greater improvements in the non-dominant side, over the training and follow-up periods. We report on a potential mechanism using global exercise interventions to facilitate improvements in reaction time and travel speed, as well as other motor control metrics, which are likely to benefit PwPD in functional daily tasks that require upper limb speed and accuracy, with lasting effects over 12 months, particularly on the non-dominant side.

## Supporting information

S1 Checklist CONSORT checklist: A point-by-point verification of the items reported in this clinical trial, ensuring compliance with CONSORT guidelines. (DOC)

S1 File Trial study protocol: A comprehensive and detailed description of the study protocol, including information on groups, interventions, and methodologies used in the study. (PDF)

S2 File MANOVA results: Additional analysis providing a head-to-head comparison, including effect sizes, F-values, degrees of freedom, and variance. (XLSX)

## References

[1] Gazewood JD, Richards DR, Clebak K. Parkinson disease: an update. Am Fam Physician 2013;87(4):267–73. 23418798

[2] Opara J, Małecki A, Małecka E, Socha T. Motor assessment in Parkinsons disease. Ann Agric Environ Med 2017;24(3):411–415. doi: 10.5604/12321966.1232774 28954481

[3] Wylie SA, Van Den Wildenberg WPM, Ridderinkhof KR, Bashore TR, Powell VD, Manning CA, et al. The effect of speed-accuracy strategy on response interference control in Parkinson’s disease. Neuropsychologia 2009;47(8–9):1844–1853. doi: 10.1016/j.neuropsychologia.2009.02.025 19428416 PMC4524649

[4] Fasano A, Mazzoni A, Falotico E. Reaching and grasping movements in Parkinson’s disease: a review. Journal of Parkinson’s disease 2022;12(4),1083–1113. doi: 10.3233/JPD-213082 35253780 PMC9198782

[5] Van Wouwe NC, Van den Wildenberg WPM, Claassen DO, Kanoff K, Bashore TR, Wylie SA. Speed pressure in conflict situations impedes inhibitory action control in Parkinson’s disease. Biological psychology 2014;101:44–60. doi: 10.1016/j.biopsycho.2014.07.002 25017503 PMC4504191

[6] Fernandez L, Huys R, Issartel J, Azulay JP, Eusebio A. Movement speed-accuracy trade-off in Parkinson’s disease. Frontiers in neurology 2018;9:897. doi: 10.3389/fneur.2018.00897 30405521 PMC6208126

[7] Sakurada T, Knoblich G, Sebanz N, Muramatsu SI, Hirai M. Probing links between action perception and action production in Parkinson’s disease using Fitts’ law. Neuropsychologia 2018;111, 201–208. doi: 10.1016/j.neuropsychologia.2018.02.001 29421296

[8] Ingram LA, Carroll VK, Butler AA, Brodie MA, Gandevia SC, Lord SR. Quantifying upper limb motor impairment in people with Parkinson’s disease: a physiological profiling approach. PeerJ 2021;9,e10735. doi: 10.7717/peerj.10735 33604177 PMC7869669

[9] Thomas M, Lenka A, Kumar Pal P. Handwriting analysis in Parkinson’s disease: current status and future directions. Movement disorders clinical practice 2017;4(6):806–818. doi: 10.1002/mdc3.12552 30363367 PMC6174397

[10] Bryant MS, Workman CD, Jamal F, Meng H, Jackson GR. Feasibility study: Effect of hand resistance exercise on handwriting in Parkinson’s disease and essential tremor. Journal of Hand Therapy 2018;31(1):29–34. doi: 10.1016/j.jht.2017.01.002 28389133

[11] Nackaerts E, Heremans E, Vervoort G, Smits‐Engelsman BC, Swinnen SP, Vandenberghe W, et al. Relearning of writing skills in Parkinson’s disease after intensive amplitude training. Movement Disorders 2016;31(8):1209–1216. doi: 10.1002/mds.26565 26990651

[12] Nackaerts E, Broeder S, Pereira MP, Swinnen SP, Vandenberghe W, Nieuwboer A, et al. Handwriting training in Parkinson’s disease: A trade-off between size, speed and fluency. PloS one 2017;12(12):e0190223. doi: 10.1371/journal.pone.0190223 29272301 PMC5741263

[13] Paul SS, Dibble LE, Olivier GN, Walter C, Duff K, Schaefer SY. Dopamine replacement improves motor learning of an upper extremity task in people with Parkinson disease. Behavioural Brain Research 2020;377,112213. doi: 10.1016/j.bbr.2019.112213 31526767 PMC7398159

[14] Ernst M, Folkerts AK, Gollan R, Lieker E, Caro-Valenzuela J, Adams A, et al. Physical exercise for people with Parkinson’s disease: a systematic review and network meta-analysis. The Cochrane database of systematic reviews 2023;1(1),CD013856. doi: 10.1002/14651858.CD013856.pub2 36602886 PMC9815433

[15] Radder DLM, Lima ALS, Domingos J, Keus SHJ, van Nimwegen M, Bloem BR, et al. Physiotherapy in Parkinson’s Disease: A Meta-Analysis of Present Treatment Modalities. Neurorehabilitation and neural repair 2020;34(10),871–880. doi: 10.1177/1545968320952799 32917125 PMC7564288

[16] Tang L, Fang Y, Yin J. The effects of exercise interventions on Parkinson’s disease: a Bayesian network meta-analysis. Journal of Clinical Neuroscience 2019;70,47–54. doi: 10.1016/j.jocn.2019.08.092 31526677

[17] Goldman JG, Volpe D, Ellis TD, Hirsch MA, Johnson J, Wood J, et al. Delivering Multidisciplinary Rehabilitation Care in Parkinson’s Disease: An International Consensus Statement. Journal of Parkinson’s disease 2024;14(1),135–166. doi: 10.3233/JPD-230117 38277303 PMC10836578

[18] Welsby E, Berrigan S, Laver K. Effectiveness of occupational therapy intervention for people with Parkinson’s disease: Systematic review. Australian occupational therapy journal 2019;66(6),731–738. doi: 10.1111/1440-1630.12615 31599467

[19] Collett J, Franssen M, Meaney A, Wade D, Izadi H, Tims M, et al. Phase II randomised controlled trial of a 6-month self-managed community exercise programme for people with Parkinson’s disease. Journal of Neurology, Neurosurgery & Psychiatry 2017;88(3):204–211. doi: 10.1136/jnnp-2016-314508 27837101

[20] Collett J, Franssen M, Winward C, Izadi H, Meaney A, Mahmoud W, et al. A long-term self-managed handwriting intervention for people with Parkinson’s disease: results from the control group of a phase II randomized controlled trial. Clinical rehabilitation 2017;31(12):1636–1645. doi: 10.1177/0269215517711232 28547999

[21] Mavrommati F, Collett J, Franssen M, Meaney A, Sexton C, Dennis-West A, et al. Exercise response in Parkinson’s disease: insights from a cross-sectional comparison with sedentary controls and a per-protocol analysis of a randomised controlled trial. BMJ open 2017;7(12):e017194. doi: 10.1136/bmjopen-2017-017194 29282259 PMC5770916

[22] Schulz KF, Altman DG, Moher D, CONSORT Group*. CONSORT 2010 statement: updated guidelines for reporting parallel group randomized trials. Annals of internal medicine 2010;152(11):726–732. doi: 10.7326/0003-4819-152-11-201006010-00232 20335313

[23] Moher D, Hopewell S, Schulz KF, Montori V, Gøtzsche PC, Devereaux PJ, et al. CONSORT 2010 explanation and elaboration: updated guidelines for reporting parallel group randomised trials. Bmj 2010;340. 10.1136/bmj.c869 PMC2844943 20332511

[24] Bohannon RW, Wang YC, Gershon RC. Two-minute walk test performance by adults 18 to 85 years: normative values, reliability, and responsiveness. Archives of physical medicine and rehabilitation 2015;96(3),472–477. doi: 10.1016/j.apmr.2014.10.006 25450135

[25] Chou KL, Stacy M, Simuni T, Miyasaki J, Oertel WH, Sethi K, et al. The spectrum of “off” in Parkinson’s disease: what have we learned over 40 years?. Parkinsonism & Related Disorders 2018;51,9–16. doi: 10.1016/j.parkreldis.2018.02.001 29456046

[26] Brooks D, Davis AM, Naglie G. Validity of 3 physical performance measures in inpatient geriatric rehabilitation. Arch Phys Med Rehabil 2006;87:105–10. doi: 10.1016/j.apmr.2005.08.109 16401447

[27] Peppe A, Ranaldi A, Chiavalon C, et al. Global Mobility Task: index for evaluating motor impairment and motor rehabilitation programs in Parkinson’s disease patients. Acta Neurol Scand 2007;116:182–9. doi: 10.1111/j.1600-0404.2007.00859.x 17714332

[28] ACSM. American College of Sports Medicine ACSM’s guidelines for exercise testing and prescription. Lippincott Williams and Wilkins, 2013. 10.1249/JSR.0b013e31829a68cf 23851406

[29] Newman DG, Pearn J, Barnes A, et al. Norms for hand grip strength. Arch Dis Child 1984;59:453–9. doi: 10.1136/adc.59.5.453 6732276 PMC1628520

[30] Cubí-Mollá P, de Vries J, Devlin N. A study of the relationship between health and subjective well-being in Parkinson’s disease patients. Value Health 2014;17:372–9. doi: 10.1016/j.jval.2014.03.002 24968997

[31] Brown CA, Cheng EM, Hays RD, et al. SF-36 includes less Parkinson disease (PD)-targeted content but is more responsive to change than two PD-targeted health-related quality of life measures. Qual Life Res 2009;18:1219–37. doi: 10.1007/s11136-009-9530-y 19714487 PMC2759458

[32] Krupp LB, LaRocca NG, Muir-Nash J, et al. The fatigue severity scale. Arch Neurol 1989;46:1121–3. 2803071 10.1001/archneur.1989.00520460115022

[33] Washburn RA, Smith KW, Jette AM, et al. The Physical-Activity Scale for the Elderly (PASE)—development and evaluation. J Clin Epidemiol 1993;46:153–62. doi: 10.1016/0895-4356(93)90053-4 8437031

[34] Da Silva TD, Ribeiro-Papa DC, Coe S, Malheiros SRP, Massetti T, Meira CM Junior, et al. Evaluation of speed-accuracy trade-off in a computer task to identify motor difficulties in individuals with Duchenne Muscular Dystrophy—A cross-sectional study. Res Dev Disabil 2020;96:103541. doi: 10.1016/j.ridd.2019.103541 31830680

[35] Fernani DCGL, Prado MTA, da Silva TD, Massetti T, de Abreu LC, Magalhães FH, et al. Evaluation of speed-accuracy trade-off in a computer task in individuals with cerebral palsy: a cross-sectional study. BMC neurology 2017;17,1–9. doi: 10.1186/s12883-017-0920-4 28750603 PMC5530971

[36] Moraes IAP, Collett J, Silva T, Monteiro CBM, Dawes H. Upper limbs fitts’ task—global exercise versus focused handwriting exercise training in Parkinson’s disease, Mendeley Data, V2, 2024. doi: 10.17632/hmzt5c3tc7.2

[37] Eldemir S, Guclu-Gunduz A, Eldemir K, Saygili F, Yilmaz R, Akbostancı MC. The effect of task-oriented circuit training-based telerehabilitation on upper extremity motor functions in patients with Parkinson’s disease: A randomized controlled trial. Parkinsonism & Related Disorders 2023;109,105334. doi: 10.1016/j.parkreldis.2023.105334 36917914

[38] Hashemi Y, Taghizadeh G, Azad A, Behzadipour S. The effects of supervised and non-supervised upper limb virtual reality exercises on upper limb sensory-motor functions in patients with idiopathic Parkinson’s disease. Human movement science 2022;85,102977. doi: 10.1016/j.humov.2022.102977 35932518

[39] Vanbellingen T, Nyffeler T, Nigg J, Janssens J, Hoppe J, Nef T, et al. Home based training for dexterity in Parkinson’s disease: a randomized controlled trial. Parkinsonism & related disorders 2017;41:92–98. doi: 10.1016/j.parkreldis.2017.05.021 28578819

[40] Mazzoni P, Hristova A, Krakauer JW. Why don’t we move faster? Parkinson’s disease, movement vigor, and implicit motivation. Journal of neuroscience 2007;27(27):7105–7116. doi: 10.1523/JNEUROSCI.0264-07.2007 17611263 PMC6794577

[41] Zhu S, Zhong M, Bai Y, Wu Z, Gu R, Jiang X, et al. The association between clinical characteristics and motor symptom laterality in patients with Parkinson’s disease. Frontiers in Neurology 2021;12: 758. doi: 10.3389/fneur.2021.663232 34135850 PMC8201506

[42] Riederer P, Jellinger KA, Kolber P, Hipp G, Sian-Hülsmann J, Krüger R. Lateralisation in Parkinson disease. Cell and tissue research 2018;373(1):297–312. doi: 10.1007/s00441-018-2832-z 29656343

[43] David FJ, Robichaud JA, Vaillancourt DE, Poon C, Kohrt WM, Comella CL, et al. Progressive resistance exercise restores some properties of the triphasic EMG pattern and improves bradykinesia: the PRET-PD randomized clinical trial. Journal of neurophysiology 2016;116(5):2298–2311. doi: 10.1152/jn.01067.2015 27582297 PMC5110637

[44] Abbruzzese G, Marchese R, Avanzino L, Pelosin E. Rehabilitation for Parkinson’s disease: Current outlook and future challenges. Parkinsonism & related disorders 2016;22:S60–S64. doi: 10.1016/j.parkreldis.2015.09.005 26360239

[45] Farley BG, Fox CM, Ramig LO, McFarland DH. Intensive amplitude-specific therapeutic approaches for Parkinson’s disease: toward a neuroplasticity-principled rehabilitation model. Topics in Geriatric Rehabilitation 2008;24(2):99–114.

[46] B Oliveira MP, M Dos Reis L, Pereira ND. Effect of resistance exercise on body structure and function, activity, and participation in individuals with Parkinson disease: a systematic review. Archives of Physical Medicine and Rehabilitation 2021;102(10):1998–2011. doi: 10.1016/j.apmr.2021.01.081 33587899

[47] Moraes Filho AV, Chaves SN, Martins WR, Tolentino GP, Homem RDCPP, de Farias GL, et al. Progressive resistance training improves bradykinesia, motor symptoms and functional performance in patients with Parkinson’s disease. Clinical Interventions in Aging 2020;15:87. doi: 10.2147/CIA.S231359 32158202 PMC6986410

[48] Hodges NJ, Lohse KR. Difficulty is a real challenge: A perspective on the role of cognitive effort in motor skill learning. Journal of Applied Research in Memory and Cognition 2020;9(4):455.

[49] Scharoun SM, Bryden PJ, Sage MD, Almeida QJ, et al. The influence of Parkinson’s disease motor symptom asymmetry on hand performance: An examination of the grooved pegboard task. Parkinson’s Disease, 2015. doi: 10.1155/2015/307474 26693383 PMC4674610

[50] Li K, Wei N, Yue S, Thewlis D, Fraysse F, Immink M, et al. Coordination of digit force variability during dominant and non-dominant sustained precision pinch. Experimental Brain Research 2015;233(7): 2053–2060. doi: 10.1007/s00221-015-4276-y 25869742

[51] Sainburg RL, Kalakanis D. Differences in control of limb dynamics during dominant and nondominant arm reaching. Journal of neurophysiology 2000;83(5):2661–2675. doi: 10.1152/jn.2000.83.5.2661 10805666 PMC10709817

[52] Sainburg RL. Convergent models of handedness and brain lateralization. Frontiers in psychology 2014;5:1092. doi: 10.3389/fpsyg.2014.01092 25339923 PMC4189332

[53] Vassiliadis P, Derosiere G, Dubuc C, Lete A, Crevecoeur F, Hummel FC, et al. Reward boosts reinforcement-based motor learning. Iscience 2021;24(7). doi: 10.1016/j.isci.2021.102821 34345810 PMC8319366

