# Supplementary material for: Sensorimotor functioning changes in response to global exercise versus handwriting upper limb exercise training in Parkinson’s disease, results from a phase II randomised controlled trial
Source: PLoS One. 2024 Aug 29;19(8):e0309217. doi: 10.1371/journal.pone.0309217 (PMC11361610; doi:10.1371/journal.pone.0309217)
Supplement: S1 File — (PDF) [file pone.0309217.s002.pdf]

Supporting Information – S2 Trial Study Protocol

INTERVENTION

Why: The exercise program was developed from previous work on community supported exercise for long-term neurological conditions.[1-3] The intervention was guided by behaviour change theory that considered an individual's capability, opportunity and motivation and incorporated appropriate evidence for safe effective exercise and self-determination theory. It aims to provide support and overcome barriers whilst encouraging self-management. A handwriting program was chosen for the control group after consultation pwP, who identified that handwriting was an important issue pwP and would have the desired effect engaging people through the intervention period. The handwriting program was developed considering the Parkinson's Disease Society guidance information sheet for handwriting.[4] Travel and gym costs and the materials required for the handwriting group were provided by the study. Both groups were instructed to perform daily activities as they usual would, including any exercises that were part of their routine before the start of the trial.

Exercise group

Where: The exercise sessions took place at community leisure facilities in Oxfordshire, Berkshire and Buckinghamshire, UK. Participants were able to choose participating facilities nearby their home to minimise travel burden. Facilities included sport centres run on behalf of local authorities, private sport centres and a specialist university facility (CLEAR unit, Oxford Brookes University). Leisure facilities had to be open to the public and have the

equipment required to deliver the required exercise content (see below). Participating facilities were aware of the research and had agreed to the use of their facilities.

Who Provided: Exercise was supported by a clinical exercise professional who was either a specialist exercise practitioner (registry of exercise professional's level 4 qualification in exercise for long term neurological conditions) or a physiotherapist. Physiotherapists working on the study received training on how to deliver the prescribed exercise in community facilities by the specialist exercise professional. Members of the leisure facility staff working in the gym were fully informed about the study and that the participants were following a prescribed exercise program.

How: The clinical exercise professional arranged and attended the first exercise session with the participant in order to introduce them to the gym environment and instruct them on the equipment and exercise program and to ensure they were competent and safe to follow the program. Thereafter participants were scheduled to receive monthly support sessions, in order to monitor and progress. The participants were able to contact the clinical exercise professional if they needed more advice and information or had any concerns. Specific details of issue encountered and strategies used are beyond the scope of this article but will be reported elsewhere.

What: The exercise program was delivered through the exercise booklet that both directed the program and acted as a diary for the participant to record their progress. An example booklet can be found at [http://www.shs.brookes.ac.uk/images/pdfs/research/movement-science/example_exercise_booklet.pdf]. The booklet also served to monitor intervention adherence and fidelity.

When and how much: The exercise programme totalled 48 sessions over a 24 week period (2x a week). Each 60minute session consisted of the following: At the start of each session, the participants performed 30 minutes of aerobic training (55-85% age predicted heart rate max (220-age)) and were able to choose from on a treadmill, bicycle ergometer, cross-trainer or rowing ergometer, depending on equipment availability. After an initial warm up of 10 minutes, participants were instructed to exercise so that heart rate was maintained for 20minutes in an aerobic training zone (Medication affecting heart rate was considered). Participants recorded the type of equipment used and actual duration, as well as perceived exertion (RPE, CR10 scale) [5] and heart rate in their training diaries. The aerobic exercise was followed by 30 minutes of resistance training. The resistance training schedule consisted of leg press, leg extensions, sit to stands, 2 arm pull down, 'wood chop' and arm raises.

Tailoring: The intervention was personalised and progressed according to the following protocols. At the initial session the exercise professional or physiotherapist set the exercise intensity so that the participants achieved 55-85 percentage age predicted maximal heart rate), for the duration of the aerobic training, participants were then taught to manipulate speed or resistance in order to maintain the exercise intensity at subsequent sessions. Initial resistance was selected so ten repetitions could be performed. For resistance exercises the clinical exercise professional instructed the participants that when two full sets of ten could be performed at a given resistance, within two minutes, to increase resistance. This would lead to a resultant decrease in repetitions and then the protocol repeated. At the monthly support session exercise intensities and progression was monitored.

Handwriting group

Where: The handwriting sessions took place in the participants own home. **Who Provided:** Handwriting was supported by the same staff that supported the exercise sessions**. How:** The clinical exercise professional went through the first work book with the participant face-to-face at the participants homes ensuring they knew how to complete the work books. As with the exercise group, thereafter participants were scheduled to receive monthly support sessions and the participants were able to contact the clinical exercise professional if they needed more advice and information or had any concerns. **What:** The program was delivered through handwriting workbooks. The workbook also served to monitor control group adherence and fidelity. An example workbook can be found at [http://www.shs.brookes.ac.uk/images/pdfs/research/movement-science/example_handwriting_booklet.pdf]. Additional equipment required was 'play dough' putty, clothes pegs (6), lolly sticks and a jar, and a soft tennis ball. **When and how much:** The handwriting program also totalled 48 sessions over a 24 week period (2x a week). The 60minute session consisted of the following, Warm up exercises for both hands (wriggling figures, making a fist, touching figure with thumb, circling wrists, shrugging shoulders and stretching hands), then a variety of writing activities (eg copying shapes, writing pangrams, writing a post card, filling in a form) and finished with hand exercises (rolling putty, using pegs, placing sticks in a jar, and ball drop and catch). Writing activities varied from workbook to workbook in order to maintain interest. **Tailoring:** There was no formal tailoring or progression, as all participants in the handwriting group followed the same workbooks. However, participants could monitor performance their own using the 'The quick brown fox jumps over the lazy dog' pangram which was performed every session and feedback was given by the clinical exercise professional at the monthly support sessions

OUTCOME MEASUREMENT

Motor Symptoms

The primary outcome measure was the **2minute walk test**.[6] Participants were asked to walk as far as they could in 2minutes along a 16m indoor walkway, turning around cones at each end; and the distance walked measured. Mobility was also assessed using the **timed up and go** test (TUG),[6] for this test participants were required to stand up from a chair, walk 3m around a cone, return to the chair and sit down as fast as they could. The time taken to complete the TUG test was recorded. Walking tests were performed once per assessment; no encouragement was given during the tests. The time taken to complete the **9 hole peg test[7]** was used to measure dexterity and was recorded as the average of 4 trials (2 dominant, 2 non dominant hand). Global motor function was assessed using the motor examination of the **MDS-UPDRS** (MDS-UPDRS-III).[8] The MDS-UPDRS-III Comprises of 14 examinations scored from 0 (absent) to 4 (severe) with a best possible score of 0 and a worse possible score of 56.

Fitness

In order to determine individual aerobic fitness (VO^2_{max}) a **stepwise incremental exercise test** was performed. Prior to the test participants for screened for safety using the Physical Activity Readiness Questionnaire (PAR-Q)[9] questionnaire and a twelve lead resting Electrocardio-gram.[10] Participants failing the screen for maximal exercise were allowed to continue in the study if they had no contraindication to the exercise intervention. The test was conducted on an electronically braked cycle ergometer (Excalibur Sport, Lode, Netherlands), integrated with a cardio pulmonary monitoring system (Metalyzer 3B, Cortex,

Germany), that controlled the work rate protocol on the ergometer and recorded breath-by-breath measurements of oxygen consumption, carbon dioxide production, ventilation and heart rate throughout the test. The work rate protocol consisted of 2minutes steps starting with unloaded cycling, then increasing to 50watts, and there after by 25watts. Whilst, the ergometer maintained a constant work load, independent of cadence, participants were instructed to aim for cadence of ~50rpm. Participants were verbally encouraged carry on for as they could and the test was terminated when the participant reached volitional exhaustion. Rate of oxygen consumption was calculated as the average oxygen consumed over the last 30secs of the test (VO^2_{max} $l.min^{-1}$).[11] **Leg extensor power** was measured using a 'leg power meter' (Medical Laboratory Workshops, Nottingham, UK)[12]. Participants sat on the leg power meter, with knee and hip flexed and were instructed to push a foot plate as hard as they could. The meter was set up so that the knee was at ~5^0 from full extension at the end of the push. The maximum power achieved from each leg separately was recorded and reported as an average of the two legs. **Grip strength** was measured using a hand held dynometer (TAKEI 5401, Takei scientific Instruments co. ltd, Niigata, Japan), whereby the participant was instructed to squeeze the handle as hard as they could and the maximum force of each hand was recorded and reported as the average of the two hands.[13]

Health and wellbeing

Health related quality of life was measured using the Euro-QOL **EQ5D-5L** [14]and **SF36,**[15, 16] scores are reported for the EQ5D-5L index score (0 to 100 visual analogue scale; 0 indicating the worst health some can imagine and 100 the best) and physical and mental SF36 scores (0 to 100 scale, with 100 indicating high health status). Non-motor symptoms

of Parkinson's disease was assessed using the Parkinson's disease **non-motor symptom questionnaire,** [17] and reported as the total score for the 30 items (symptoms) of questionnaire with 1 scored if a symptom is has been experienced during the past month. Self-reported fatigue was measured using the **Fatigue Severity Scale** (FSS) (average score from 9 statements rated 1 to 7, with 7 indicating greatest impact of fatigue).[18] Health status was measured using **Body Mass Index** (BMI = Weight (kg)/ (height(m) ^{2}), resting **blood pressure** (Mean arterial pressure = (Systolic blood pressure (mmHg) + 2 x Diastolic blood pressure(mmHg) / 3)) and physical activity using the **Physical Activity Scale for the Elderly** (PASE)[19]. The PASE measures level of self-reported physical activity recalled over the previous 7 days with a higher score indicating more activity.

REFERENCES

1. Elsworth C, Dawes H, Sackley C , et al. A study of Perceived facilitators to physical activity in neurological conditions. *Int J Ther Rehabil* 2009;16(1):17 - 24
2. Winward C, C and the LIFE group. Supporting community-based exercise in long-term neurological conditions: experience from the Long-term Individual Fitness Enablement (LIFE) project. *Clin Rehabil* 2011;25(7):579-87
3. Elsworth C, Winward C, Sackley C, et al. Supported community exercise in people with long-term neurological conditions: a phase II randomized controlled trial. *Clin Rehabil* 2011;25(7):588-98
4. Parkinsons Disease Society. *Handwriting and Parkinson's* 2008 , PD UK
5. Borg, G.A., *Borg's preceived exertion and pain scales*. 1998, Leeds: Human Kinetics.
6. Brooks, D., A.M. Davis, and G. Naglie, Validity of 3 physical performance measures in inpatient geriatric rehabilitation. *Arch Phys Med Rehabil*, 2006. **87**(1): p. 105-10.

7. Balcer LJ. Clinical outcome measures for research in multiple sclerosis. *J Neuroophthalmol* 2001;21(4):296-301

8. Peppe A, Ranaldi A, Chiavalon C, et al. Global Mobility Task: index for evaluating motor impairment and motor rehabilitation programs in Parkinson's disease patients. *Acta Neurol Scand* 2007;116(3):182-9

9. Bredin, S.S., et al., PAR-Q+ and ePARmed-X+: new risk stratification and physical activity clearance strategy for physicians and patients alike*. Can Fam Physician*, 2013. **59**(3): p. 273-7.

10. Thompson, P.D., et al., ACSM's new preparticipation health screening recommendations from ACSM's guidelines for exercise testing and prescription, ninth edition*. Curr Sports Med* Rep, 2013. **12**(4): p. 215-7.

11. ACSM. *American College of Sports Medicine ACSM's Guidelines For Exercise Testing And Prescription* Lippincott Williams and Wilkins, 2013.

12. Smith CE, Dawes H, Collett J, et al. A pilot investigation into the relationship between leg power and walking speed in individuals with acquired brain injury. *Physiotherapy* 2005;9(1)49-60

13. Newman DG, Pearn J, Barnes A, et al. Norms for hand grip strength. *Arch Dis Child* 1984;59(5):453-9

14. Cubi-Molla P, de Vries J, Devlin N. A study of the relationship between health and subjective well-being in Parkinson's disease patients. *Value Health* 2014;17(4):372-9

15. Hawthorne, G., et al., *The SF36 Version 2: critical analyses of population weights, scoring algorithms and population norms.* Qual Life Res, 2007. **16**(4): p. 661-73.

16. Brown CA, Cheng EM, Hays RD, et al. SF-36 includes less Parkinson Disease (PD)-targeted content but is more responsive to change than two PD-targeted health-related quality of life measures. *Qual life res* 2009;18(9):1219-37

17. Chaudhuri KR, Martinez-Martin P, Schapira AH, et al. International multicenter pilot study of the first comprehensive self-completed nonmotor symptoms questionnaire for Parkinson's disease: the NMSQuest study. *Mov Disord* 2006;21(7):916-23 d

18. Krupp LB LN, Muir-Nash J, Steinberg AD. The Fatigue Severity Scale. *Arch Neurol* 1989;46:1121-23

19. Washburn RA, Smith KW, Jette AM, et al. The Physical-Activity Scale for the Elderly (Pase) - Development and Evaluation. *J Clin Epidemiol*. 1993;46(2):153-62
